# Multi-cell type human liver microtissues for hepatotoxicity testing

**DOI:** 10.1007/s00204-012-0968-2

**Published:** 2012-11-11

**Authors:** S. Messner, I. Agarkova, W. Moritz, J. M. Kelm

**Affiliations:** InSphero AG, Technoparkstrasse 1, 8005 Zurich, Switzerland

**Keywords:** 3-dimensional, Spheroids, Kupffer cells

## Abstract

Current 2-dimensional hepatic model systems often fail to predict chemically induced hepatotoxicity due to the loss of a hepatocyte-specific phenotype in culture. For more predictive in vitro models, hepatocytes have to be maintained in a 3-dimensional environment that allows for polarization and cell–cell contacts. Preferably, the model will reflect an in vivo-like multi-cell type environment necessary for liver-like responses. Here, we report the characterization of a multi-cell type microtissue model, generated from primary human hepatocytes and liver-derived non-parenchymal cells. Liver microtissues were stable and functional for 5 weeks in culture enabling, for example, long-term toxicity testing of acetaminophen and diclofenac. In addition, Kupffer cells were responsive to inflammatory stimuli such as LPS demonstrating the possibility to detect inflammation-mediated toxicity as exemplified by the drug trovafloxacin. Herewith, we present a novel 3D liver model for routine testing in 96-well format capable of reducing the risk of unwanted toxic effects in the clinic.

**Electronic supplementary material:**

The online version of this article (doi:10.1007/s00204-012-0968-2) contains supplementary material, which is available to authorized users.

## Introduction

Current strategies to test drug-induced liver injury (DILI) are predominantly based on in vivo animal models (Hartung 2009). However, significant species-specific variation between rodents and humans as well as genetic variability in humans impacts the extrapolation to the clinical situation (Hartung 2009). A recent analysis demonstrated that 43 % of toxic effects in humans were correctly predicted by tests in rodents, whereas this increased to 63 % when non-rodent animals were included (Olson et al. 2000). This low correlation highlights the fact that many adverse effects are not detected by traditional in vivo toxicity tests. More organotypic human in vitro models are expected to support toxicity assessment and decrease the risk of DILI in the clinic. Unfortunately, maintaining liver-specific functionality in vitro is a delicate business as hepatocytes have to retain their polarized 3D structure to maintain liver-specific functionality (Lecluyse et al. 2012; Berthiaume et al. 1996). Growing a single layer of hepatocytes between two extracellular matrix layers is the current gold standard method to maintain polarization. However, such hepatocyte cultures are phenotypically and functionally not very stable over time which impedes their use for long-term toxicity testing (Berthiaume et al. 1996). Furthermore, hepatocyte sandwich cultures are difficult to scale down to a 96-well format due to the instability of the overlaying gels and pronounced edge effects. For these reasons, larger well plates are typically used which hampers toxicity testing at early time points in the drug development process.

Primary mammalian cells retain their capacity to reform a tissue without the use of any scaffold material. Gravity-enforced cellular self-assembly in hanging drops is a well-established technology for tissue reformation enabling the formation of size-controlled, multi-cell type microtissues (Kelm and Fussenegger 2004). Assembling primary human hepatocytes into 3D liver microtissues allows cells to maintain extensive cellular contacts. Heterotypic cell–cell contacts in co-cultures further enhance the hepatocellular phenotype, maintaining hepatocytes in their differentiated state (Lecluyse et al. 2012). In addition, the implementation of non-parenchymal cells provides hepatocytes with diffusible growth factors and cytokines. For example, Kupffer macrophages release both pro-proliferative (e.g., TNF-α, IL-6) and anti-proliferative (IL-1, TGF-β) cytokines and signals (Lecluyse et al. 2012). These cytokines were shown to be involved in precipitating idiosyncratic toxicity of certain drugs, such as trovafloxacin (Liguori et al. 2010; Shaw et al. 2007, 2010). Treatment of mice or rats with inflammatory stimuli such as LPS or TNF-α together with trovafloxacin caused toxicity only in the presence of the inflammatory stimulus. However, routine assessment of inflammation-mediated toxicity in vitro has so far been difficult due to lack of commercially available primary human liver model systems incorporating inflammatory cells.

## Results and discussion

**Fig. 1:**
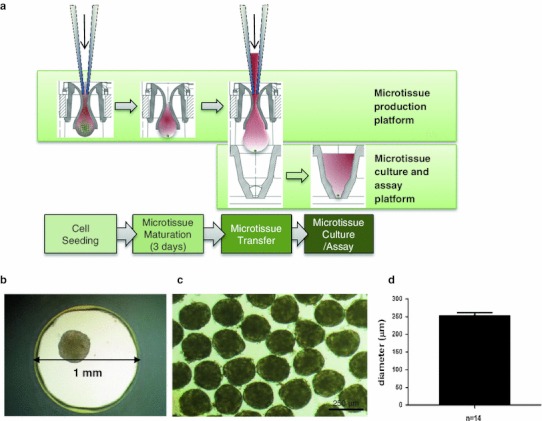
Liver microtissue production and culture. **a** Liver microtissues were produced in a 96-well hanging-drop culture platform (Gravity PLUS™). After microtissue formation, they were transferred into a microtissue-specific 96-well culture and assay platform (Gravity TRAP™). Further maintenance and compound treatments were performed in Gravity TRAP™ plates (3D Insight™ Human Liver Microtissues). **b** Bright field microscopy of a human liver microtissue. **c** Bright field microscopy of pooled human liver microtissues. **d** Size profiling of human liver hepatospheres shown in (**c**) [253 ± 7.4 μm in diameter (*n* = 14)]

**Fig. 2:**
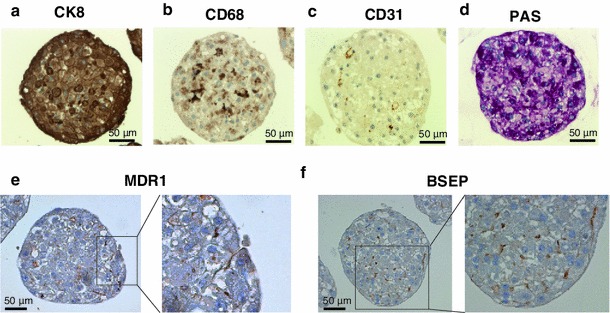
Morphological characterization of liver microtissues. **a** Immunohistochemistry (IHC) staining for CK8 (epithelial cell marker). **b** IHC staining for CD68 (Kupffer macrophage marker). **c** IHC staining for CD31 (endothelial cell marker). **d** Periodic acid schiff staining (PAS) indicates distributed glycogen storage within hepatosphere. **e** IHC staining for multidrug resistance protein 1 (MDR1) indicates polarized expression of MDR1 on hepatocyte membranes, suggesting the formation of bile canaliculi**. f** IHC staining for bile salt export pump (BSEP) verifying bile canaliculi formation within hepatosphere

**Fig. 3:**
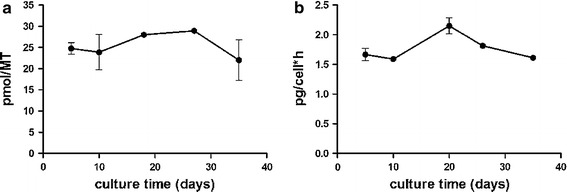
Hepatosphere viability and functionality over 5 weeks in culture.** a** Intra-tissue ATP quantification. ATP content per microtissue is depicted (pmol ATP/MT) as an indicator of cell viability and vitality. **b** Quantification of secreted albumin by ELISA over time, normalized to the initial hepatocyte cell number and time

Here, we introduce a human liver microtissue model in a 96-well format composed of cryopreserved primary human hepatocytes in combination with non-parenchymal cells (Kupffer and endothelial cells) and its use for long-term testing and inflammation-mediated toxicity (3D Insight™ Human Liver Microtissues). The accumulation of hepatocytes and non-parenchymal cells in hanging drops resulted in microtissue formation within 3 days (Fig. 1a). After microtissue formation, the spheroids were either harvested for histological analysis or transferred into a non-adhesive spheroid-specific 96-well plate for long-term culture and drug treatment (Fig. 1b–d). Immunohistochemical staining for the epithelial marker cytokeratins 8 (CK8) reveals an intact cellular phenotype, indicates direct cell–cell contacts and the typical polygonal, bicuboidal shape of hepatocytes (Fig. 2a). Kupffer cell populations were distributed throughout the microtissue and were observed by CD68 staining similar to endothelial cells positive for CD31 (Fig. 2b, c). The macrophages exhibited typical morphology with elongated shapes. Glycogen storage capability was confirmed by periodic acid schiff staining (Fig. 2d, dark violet stain). The presence of transporters was exemplified by staining for the multidrug resistance protein 1 (MDR1) and bile salt export pump (BSEP) (Fig. 2e, f). These transporters are ATP-dependent drug efflux pumps mediating transport of endogenous and xenobiotic substances. The transporters are clearly expressed in a polarized manner on the apical surface of the primary hepatocytes (Fig. 2e, f). Their staining pattern indicates presence of bile canaliculi, into which hepatocytes secrete their metabolized toxic products. Some of the bile canaliculi appear to be open to the outer surface of the hepatosphere, as highlighted by MDR1 staining (Fig. 2e). Liver microtissues remained stable over 5 weeks in culture as shown by a constant ATP content (Fig. 3a). This extended life span compared to 2D cultures of hepatocytes is most likely due to extensive cell–cell contacts, which are essential for maintaining the differentiated status of hepatocytes. Besides the stable viability, functionality of liver microtissues is preserved over 5 weeks, as indicated by persistent albumin secretion (Fig. 3b).

**Fig. 4:**
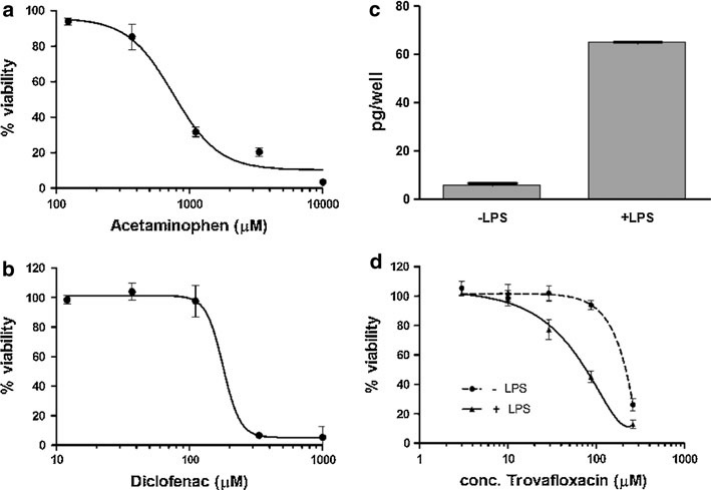
Long-term toxicity and inflammation-mediated toxicity testing with human liver microtissues using intra-tissue ATP as endpoint measurement. **a** Dose–response of acetaminophen toxicity after 14 days treatment (3 re-dosing) resulted in an IC_50_ value of 754.2 μM. **b** Dose–response curve of diclofenac supplemented for 14 days (3 re-dosing) resulted in an IC_50_ value of 178.6 μM. **c** Quantification of IL-6 secretion with ELISA measurement. Hepatosphere was induced for 48 h with 10 μg/ml LPS. Induction with LPS let to a tenfold increase in IL-6 secretion. **d** Dose–response of trovafloxacin induced toxicity in presence and absence of LPS. Presence of LPS decreased the IC_50_ threefold from 220 (-LPS) to 71 μM (+LPS)

The prolonged hepatocyte lifetime and functionality in comparison with conventional 2D culture of hepatocytes allows for long-term studies with repeated dosing to evaluate chronic hepatotoxic effects. Two hepatotoxic compounds acetaminophen and diclofenac were tested with respect to their long-term toxicological profile. Acetaminophen is the major cause of DILI in humans, although toxicity is dose-dependent and varies between patient populations (Stine and Lewis 2011). At therapeutic doses, a proportion of the drug undergoes bio-activation by CYP2E1, CYP1A2 and CYP3A4. The reactive intermediate depletes intracellular glutathione pools leading to hepatocyte cell death (Park et al. 2005). So far, 2D cultures of hepatocytes have not been able to convincingly recapitulate acetaminophen-induced toxicity in vitro (Fey and Wrzesinski 2012). Treatment of liver microtissues over 14 days with 3 re-dosing’s resulted in a concentration-dependent increasing cell death with an IC_50_ value of 754.2 μM (Fig. 4a). Diclofenac is a non-steroidal anti-inflammatory drug that has a strong association with hepatotoxicity. The mechanism is thought to involve phase I enzyme activity (multiple P450-catalyzed oxidations), phase II enzyme activity (glucoronylation) and mechanism-based inhibition (Park et al. 2005). In comparison with 2D cultures of human hepatocytes (calculated IC_50_ value of 331 μM) (Bort et al. 1999), long-term treated liver microtissues displayed an increased sensitivity toward this drug with an IC_50_ value of 178.6 μM (Fig. 4b).

Most directly hepatotoxic compounds are detected during pre-clinical investigations. However, indirectly hepatotoxic compounds involving the immune system are not detected during pre-clinical phases, such as trovafloxacin (Shaw et al. 2010). Recent animal experiments indicated that trovafloxacin is only hepatotoxic in combination with an inflammatory stimulus, such as lipopolysaccharide (LPS) or TNF-α (Shaw et al. 2007, 2010; Liguori et al. 2010). The mechanism is thought to involve enhanced cytokine secretion and accumulation in the liver, causing caspase activation and subsequent liver injury. Induction of the inflammatory response in liver microtissues by LPS resulted in elevated levels of IL-6 secretion, verifying the responsiveness of incorporated macrophages in the liver microtissues (Fig. 4c). The addition of LPS shifted the hepatotoxic threshold of trovafloxacin about threefold from 220 (without LPS) to 71 μM in the presence of LPS (Fig. 4d).

Developed to overcome the limitations of conventional 2D culture, multi-cell type 3D liver microtissues resemble liver-like cell composition and an extended stability in culture. The long-term viability and functionality of liver microtissues allows for routine compound testing as well as chronic and inflammation-mediated toxicity. The 96-well format allows for microtissue mass production enabling the implementation of an organotypic liver model at an early time point in drug development.

## Electronic supplementary material

Below is the link to the electronic supplementary material.

Supplementary material 1 (DOCX 21.2 kb)
