# Supplementary material for: Multi-cell type human liver microtissues for hepatotoxicity testing
Source: Arch Toxicol. 2012 Nov 11;87(1):209–13. doi: 10.1007/s00204-012-0968-2 (PMC3535351; doi:10.1007/s00204-012-0968-2)
Supplement: Supplementary file 1 — Supplementary material 1 (DOCX 21.2 kb) [file 204_2012_968_MOESM1_ESM.docx]

**Supplementary Material**

*Multi-cell type human liver microtissues for hepatotoxicity testing*

# Material and Methods

## Cell culture and hepatosphere formation

Cryopreserved primary human hepatocytes and non-parenchymal cell population were obtained from Celsis-IVT. A combination of hepatocytes and NPCs were seeded in a 96-well hanging drop culture platform (GravityPLUS^TM^, InSphero AG, Zurich, Switzerland) in liver tissue formation medium (InSphero AG). After tissue-formation, the hepatospheres (human liver microtissues) were transferred to a spheroid-specific 96-well microtissue receiver plate (GravityTRAP^TM^, InSphero AG). Hepatospheres were further cultivated in the GravityTRAP^TM^ plates with a tissue-specific serum-free maintenance medium (LIMM, InSphero AG). Medium exchanges were performed twice a week unless otherwise indicated.

## Immunohistochemistry

Hepatospheres were harvested after 3 days re-aggregation in the hanging drop plate into 1.5 ml tubes, washed with 1 ml PBS-buffer and fixated with 4% PFA for 2 hours at room temperature. Specimens were further processed for paraffin embedding and immunohistological staining. Sections were stained for CK8 (Becton Dickinson Immunocytometry Systems, Cat No. 345779), CD68 (Novocastra Laboratories Ltd, Cat No. NCL-L-CD68), CD31 (Abcam Limited, Cat. No. ab28364) and PAS (stained by Sophistolab AG, Eglisau, Switzerland), MDR1 (CHEMICON international, Inc., Cat No. MAB4120) and BSEP (stained at the Institute of Pathology, University hospital Zurich, Zürich, Switzerland).

## Albumin and IL-6 ELISA

Quantification of secreted human albumin was performed on supernatants of hepatospheres 24 hours after the last medium exchange at indicated time points. ELISA was performed according to manufacturer’s protocol (Bethyl-laboratories, Montgomery, USA; Catalog Nr.: E88-129). Quantification of Interleukin-6 (IL-6) secretion according to the manufacturer’s instructions (Life Technologies, USA, Catalog Nr.: KHC0061). Supernatants of hepatospheres were taken after 48 hours induction with 10µg/ml Lipopolysaccharide (Sigma Aldrich, Buchs, Switzerland; Catalog Nr.: L6529). Fluorescence was measured with a Tecan Infinite M200Pro multiplate reader (Tecan Group Ltd., Männedorf, Switzerland).

## Compound treatment

Acetaminophen, diclofenac and trovafloxacin were obtained from Sigma-Aldrich (Catalog Nr: A7302; D6899, PZ0015). Acetaminophen and diclofenac were dissolved in LIMM-Medium at top concentrations of 10 mM resp. 1 mM and further diluted for 1:3 for compound treatments at indicated concentrations. Trovafloxacin was dissolved in DMSO and further diluted at indicated concentrations with a final DMSO concentration of 1%. Acetaminophen and diclofenac treatments were performed over 14 days with 3 re-dosing’s. Trovafloxacin was applied as single dose with or without 10 µg/ml LPS (Sigma Aldrich, Buchs, Switzerland; Catalog Nr.: L6529) and incubated for 5 days (corresponding to day 6-11 of culture).

## ATP-assay

Measurement of cellular viability of hepatospheres was performed with CellTiter-Glo® reagent (Promega, Madison, USA). The assay was performed according to manufacturer’s protocol with an increased incubation time of 20 minutes. Luminescence was quantified with the Tecan Infinite M200Pro (Tecan Group Ltd., Männedorf, Switzerland).
